# Application of rebound tonometer on skin surface pressure measurement: a pilot proof of concept study 

**DOI:** 10.3389/fbioe.2025.1662361

**Published:** 2025-11-21

**Authors:** Weiwei Bian, Wei Ding, Lan Yang, Ying Wu, Ying Huang

**Affiliations:** 1 Department of Nursing, Shanghai Ninth People’s Hospital, Shanghai Jiao Tong University School of Medicine, Shanghai, China; 2 Department of Plastic and Reconstructive Surgery, Shanghai Ninth People’s Hospital, Shanghai Jiao Tong University School of Medicine, Shanghai, China

**Keywords:** skin tissue expansion, dilator, pressure measurement, rebound tonometer, children

## Abstract

**Background:**

Skin tissue expansion is a widely used technique for generating additional healthy tissue to reconstruct congenital or acquired defects. Successful expansion relies on appropriate pressure management, since insufficient or excessive pressure may lead to prolonged treatment or expansion failure. Previous studies have attempted to estimate mechanical stress on expanded skin by measuring intracapsular pressure; however, this approach does not directly capture true mechanical pressure at specific skin locations. A direct method for measuring skin surface pressure remains unavailable. In this study, we evaluated the feasibility of using a rebound tonometer–a device designed for intraocular pressure measurement–for direct, site-specific assessment of skin surface pressure and for monitoring adverse events during tissue expansion.

**Methods:**

Patients with giant nevi meeting the eligibility criteria were enrolled following dilator implantation. Surface pressure measurements were obtained using the Icare® TA01i tonometer at nine predefined sites on the expanded region. The expansion protocol involved incremental saline injections of approximately 10% of the dilator’s total volume per session. At each site, pressure was recorded at three timepoints: pre-inoculation, immediately post-inoculation, and 30 min post-inoculation. Monitoring continued throughout the expansion process until the dilator reached twice its original volume.

**Results:**

A total of five pediatric patients (2 boys and 3 girls), aged 3–6 years, were enrolled in this study. Each patient underwent a 34-day expansion period comprising eight or ten saline injections. No adverse events were recorded throughout the study. The rebound tonometer successfully detected dynamic changes in skin surface pressure corresponding to dilator volume increases. Furthermore, multi-point measurements across the expanded region confirmed the presence of spatially heterogeneous pressure distribution, demonstrating significant variations between different anatomical locations.

**Conclusion:**

This pilot study supports the technical feasibility of using a rebound tonometer for monitoring skin surface pressure dynamics during tissue expansion. The device demonstrated favorable operational efficiency, safety profile, and measurement reproducibility throughout the expansion period. These preliminary findings suggest that rebound tonometry may represent a promising monitoring approach in tissue expansion protocols, though larger-scale validation is warranted to substantiate these observations.

## Introduction

Skin and soft tissue expansion is a reconstructive surgical technique that mechanically stretches skin and soft tissue to generate additional healthy tissue for repairing congenital or acquired defects (Liu and Ma, 2019). In this procedure, a temporary tissue dilator is implanted subcutaneously. The internal pressure exerted by the dilator gradually stretches the overlying soft tissue, facilitating donor site expansion. Appropriate pressure management is critical: insufficient expansion prolongs treatment duration and increases risks of complications such as secondary infection and seroma, while overexpansion may lead to flap necrosis (Chen and Ma, 2019; Patel et al., 2014).

Intracapsular pressure or volume has been used as an indirect indicator of the mechanical force acting on expanded tissues (Xu et al., 2013). However, due to uneven adherence between the dilator and surrounding tissue, along with heterogeneous pressure distribution across the expanded region, intracapsular measurements often fail to reflect true localized mechanical conditions. This inaccuracy can result in suboptimal expansion—either insufficient or excessive—potentially causing adverse outcomes and compromising surgical success.

The Icare® TA01i rebound tonometer, originally developed for intraocular pressure measurement (Kontiola, 1997), offers a potential solution for direct surface pressure assessment. Its operation involves a lightweight probe propelled by a spring mechanism; upon contact with the surface, the deceleration time—inversely correlated with pressure—is recorded. This mechanism enables precise, site-specific measurement, suggesting its potential applicability in monitoring mechanical forces during tissue expansion.

### Study aim

This study aimed to assess the capability of a rebound tonometer in measuring surface pressure at various expansion site locations.

## Methods

### Study design and population

The study population comprised pediatric patients undergoing dilator implantation for forehead reconstruction. Written informed consent was obtained from all guardians prior to enrollment. The forehead was selected as the exclusive implantation site due to its distinctive anatomical characteristics: the thin subcutaneous layer with minimal adipose tissue results in consistently high skin tension across pediatric age groups, while the underlying frontal bone provides stable structural support for the expanded skin.

The expansion protocol targeted a final volume between 1 and 2 times the original dilator capacity. During initial filling phases, fluid dynamics within the dilator led to shape instability until reaching the designated baseline volume (1×). Upon achieving this baseline, the dilator maintained a stable configuration, constraining internal fluid movement and ensuring consistent skin pressure during patient movement. However, based on clinical experience, expansion beyond twice the original volume was avoided due to significantly increased risk of device rupture.

### Inclusion criteria

Patients were enrolled according to the following criteria:

1. Age between 3 and 6 years;
2. Placement of tissue dilator in the forehead region;
3. Demonstrated ability to cooperate with medical procedures;
4. Absence of significant congenital comorbidities, including cardiovascular disorders or neoplasms.

### Exclusion criteria

Patients were excluded from participation based on the following:

1. Age below 3 years or beyond preschool age range;
2. Dilator placement in non-forehead locations (e.g., limbs or trunk), where differences in subcutaneous fat distribution and skin tension would introduce confounding biomechanical variables;
3. Development of intervening congenital conditions during the study period.

### Measurement

Subjects were seated in a comfortable position with the head and torso maintained upright. Pressure measurements were recorded at three time points: before saline inoculation, immediately after inoculation, and 30 min post-inoculation.

**FIGURE 1:**
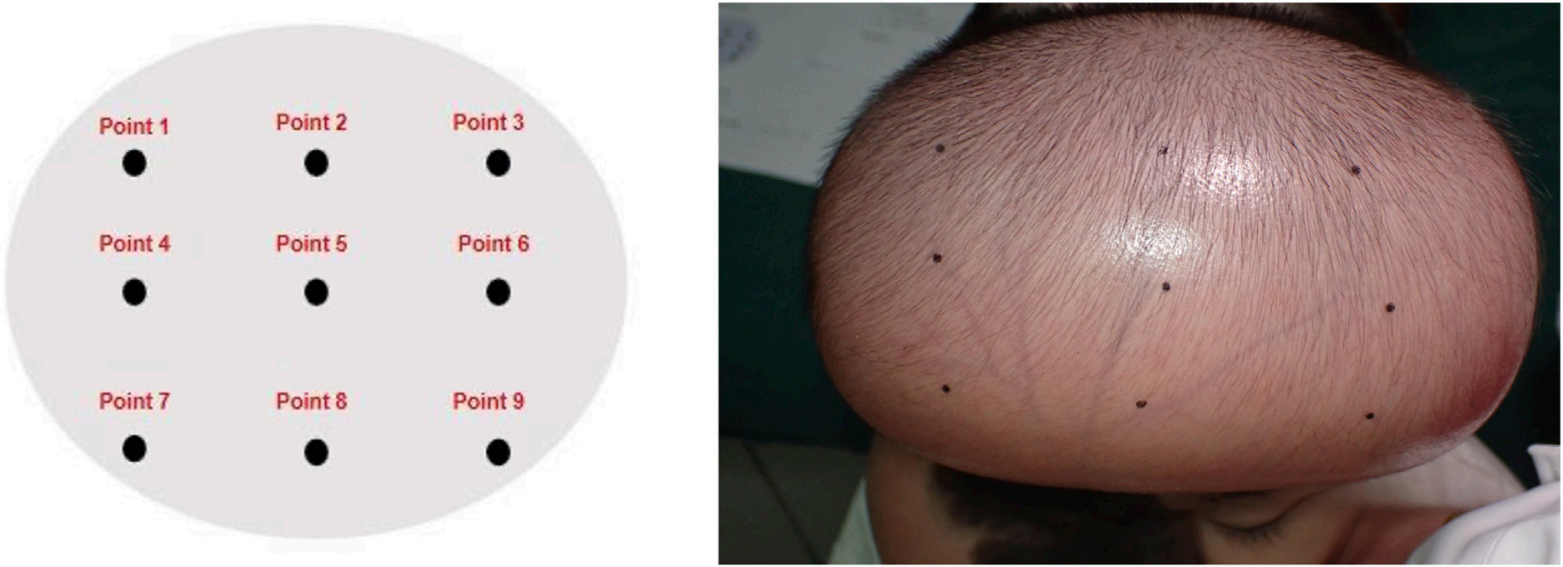
Illustration of the measurement sites. Selection of measurement sites was guided by key criteria including palpable firmness, comprehensive surface coverage, and avoidance of hair-bearing areas.

Measurement sites were identified by treating the expanded flap surface as a planar region. Sites were selected based on three criteria: palpable firmness (indicating higher pressure), broad surface coverage, and avoidance of hair-bearing areas (Figure 1).

The Icare® TA01i tonometer (Finland) was used for all measurements. The device was activated by pressing the measurement button, indicated by a “00” display, signaling readiness. Once initialized, the probe remained magnetized without descending.

**FIGURE 2:**
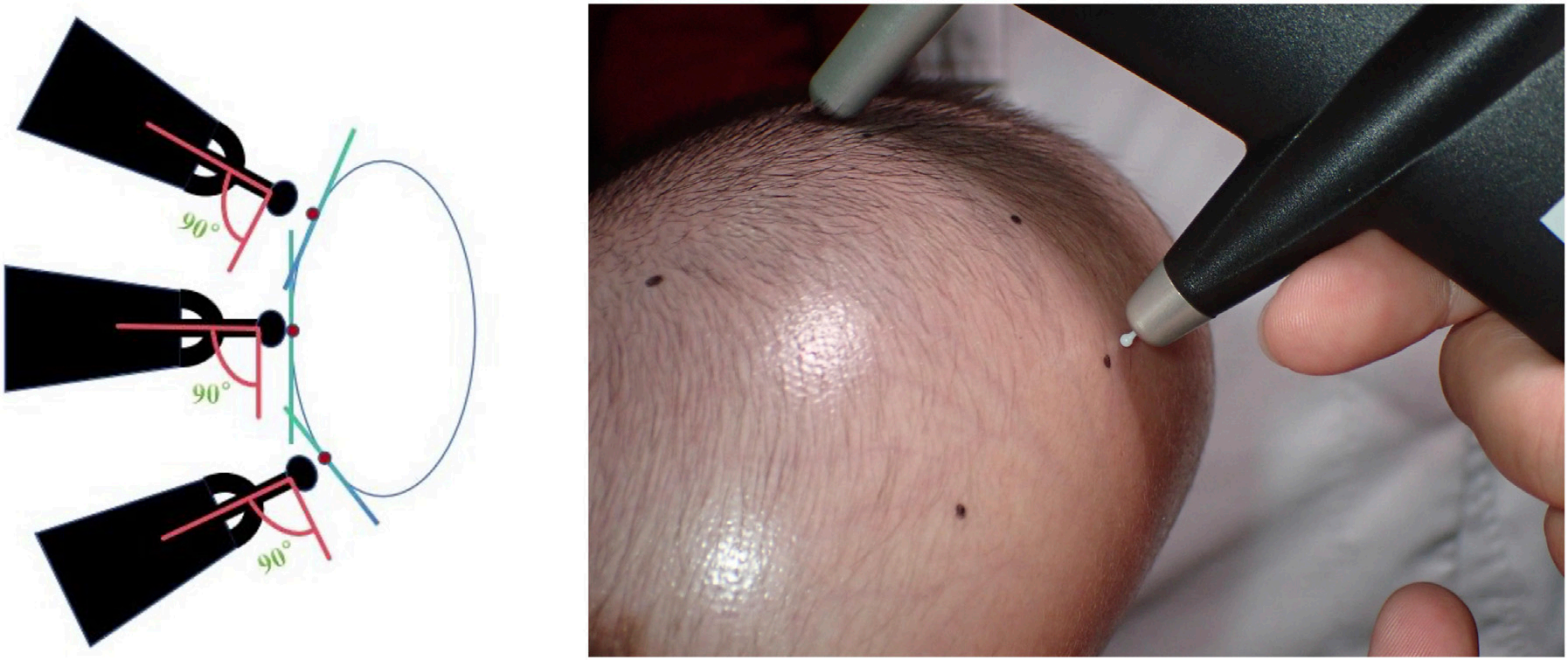
Measurement of Skin Surface Pressure with the Icare® TA01i Tonometer Probe positioned perpendicularly to the measurement site for six consecutive measurements per locus.

Operators held the tonometer in the right hand, while the left hand grasped the right wrist to form a triangular forearm structure for stabilization. The probe was positioned perpendicular to the measurement site without applying additional pressure. Six consecutive measurements were performed per site by tapping the measurement button. Results differing by no more than 0.5 mmHg were averaged to determine the final surface pressure value (Figures 1, 2).

### Data collection tool

Two investigators conducted all measurements at each time point. One investigator, specifically trained in tonometer operation, performed all measurements using standardized positioning and techniques. The second investigator promptly recorded the tonometer output, which represented the mean value of six consecutive measurements automatically calculated by the device. Following each recording session, both investigators jointly verified the data to ensure accuracy and consistency.

## Results

In this study, five pediatric patients (2 boys and 3 girls) with giant congenital nevi were enrolled, each receiving a single implanted tissue dilator. The demographic and clinical characteristics of the participants are summarized in the Table 1. During each expansion session, saline was injected at a volume corresponding to approximately 10% of the total dilator capacity. Skin surface pressure was measured as the dilator volume increased from baseline to twice its original size, a process involving eight or ten inflation sessions over a total duration of 34 ± 1.73 days.

**TABLE 1:** Demographic characteristics of participants.

Cases	Age	Duration of inoculation	Implantation	Volume of dilator	Final volume of dilator	Duration from 1 to 2 time(s)	Rate of perinoculation of dilator volume	Cycle of inoculation
Case1	4	97 days	Forehead	300 mL	960 mL	34 days	10%	Twice a week
Case2	4	135 days	Forehead	400 mL	1680 mL	34 days	10%	Twice a week
Case3	4	90 days	Forehead	350 mL	999 mL	33 days	10%	Twice a week
Case4	5	87 days	Forehead	400 mL	1185 mL	34 days	10%	Twice a week
Case5	4	85 days	Forehead	300 mL	850 mL	30 days	13%	Twice a week

**FIGURE 3:**
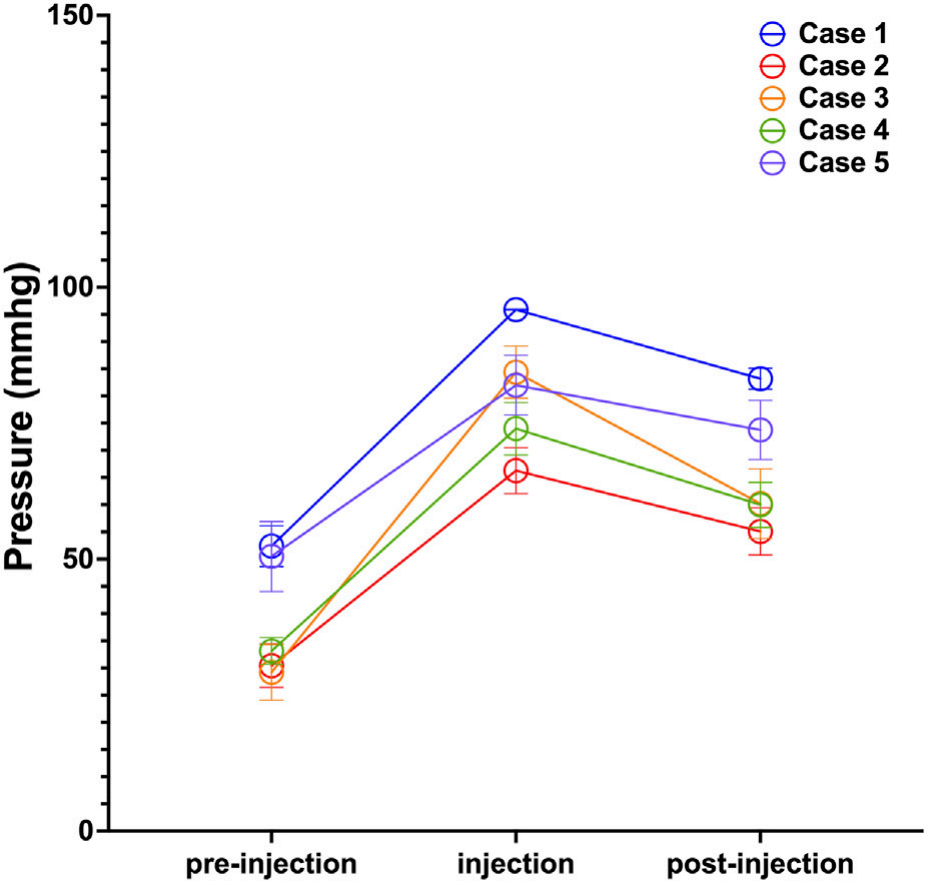
Pressure Dynamics Following Saline Injection at Site 5. Measurements were taken before injection (pre-injection), immediately post-injection (injection), and 30 min post-injection (post-injection). Data points represent the mean pressure across all saline injections; error bars indicate standard deviation.

Surface pressure was assessed at nine predefined loci covering the expanded skin area, with detailed results provided in the Supplementary Table. To illustrate temporal dynamics, pressure readings from site 5 across all five subjects are plotted in Figure 3. The Icare® TA01i tonometer successfully captured pressure changes following saline injection, consistently revealing a “climb–drop” pattern. For instance, in Case 1, the skin surface pressure at site 5 increased from a baseline of 52.40 ± 11.87 mmHg to a peak of 95.90 ± 0.32 mmHg immediately after injection, reflecting the direct mechanical stress induced by dilator inflation. Within 30 min post-injection, the pressure decreased to 83.20 ± 6.09 mmHg, indicative of tissue relaxation and redistribution of the dilator volume into adjacent areas—a process attributable to both skin recoil and the biomechanical accommodation of peripheral tissues.

**FIGURE 4:**
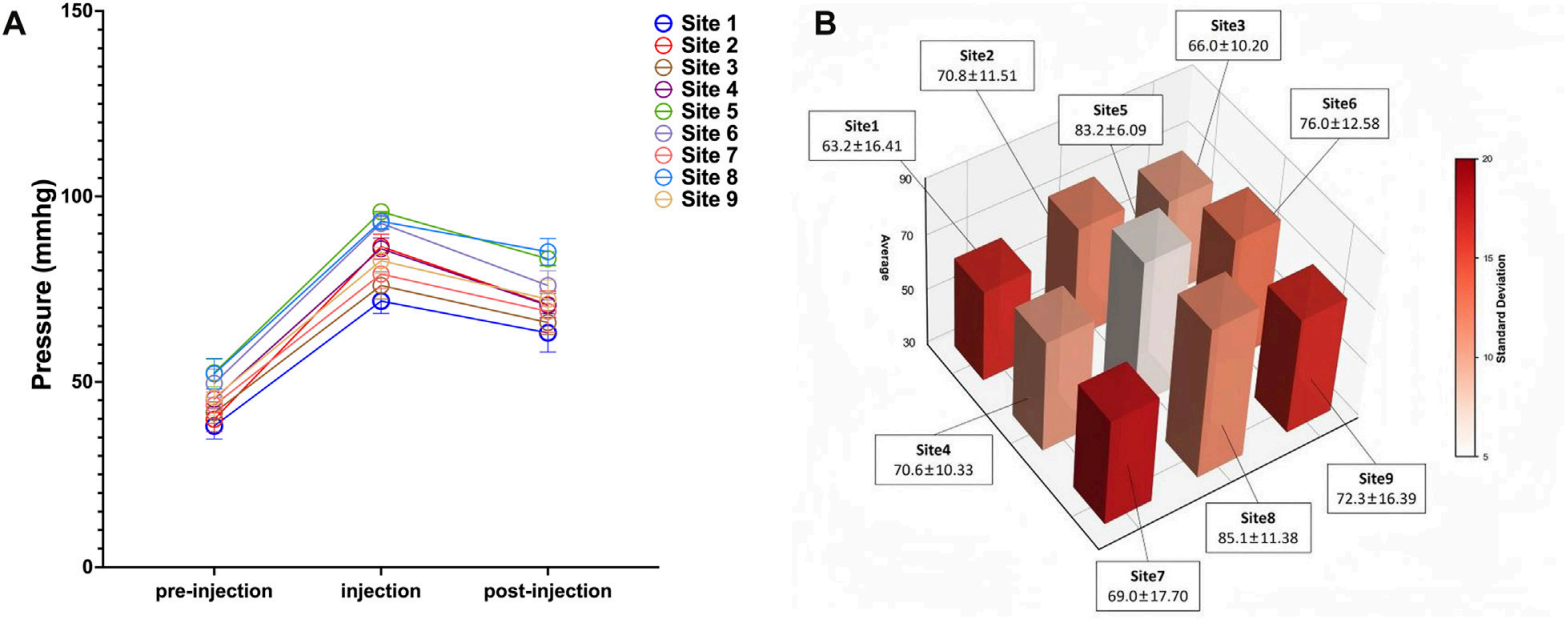
Spatial Heterogeneity in Pressure Changes after Saline Injection (Case 1). **(A)** Skin surface pressure dynamics at all measurement sites following saline injection; **(B)** Spatiotemporal pattern of skin surface pressure at all monitored sites. Data points represent the mean pressure across all saline injections; error bars indicate standard deviation.

Notably, pressure reduction did not follow a symmetrical gradient from the expansion center. As illustrated in Figure 4A for Case 1, despite sites 1 and 9 being approximately equidistant from site 5, their recorded pressures were 63.20 ± 16.41 mmHg and 72.30 ± 16.39 mmHg, demonstrating marked spatial heterogeneity. Furthermore, the pressure decline was less pronounced on the side encompassing sites 7–9 compared to that of sites 1–3, suggesting regional differences in tissue elasticity or resistance to expansion (Figure 4B). These variations underscore how local skin compliance and the mechanical properties of surrounding tissues influence pressure distribution.

Collectively, these findings underscore that pressure distribution across the expanded skin is non-uniform and influenced by localized tissue properties. Despite the limited sample size, the results support the potential utility of the Icare® TA01i tonometer for monitoring biomechanical changes during tissue expansion.

## Limitations

This preliminary study demonstrates the feasibility of using the Icare® TA01i rebound tonometer for monitoring skin surface pressure during skin expansion. However, the findings are subject to two main limitations. Firstly, the exclusive focus on forehead reconstruction limits the generalizability of the results. The biomechanical environment of the forehead, with skin directly overlying the rigid cranial bone, is anatomically unique and may not reflect the expansion dynamics in other body regions supported by softer tissues. Secondly, the small cohort of only five patients considerably restricts the external validity of our observations. The limited sample size prevents robust conclusions about the broader applicability of this monitoring technique. Future studies involving larger, multi-anatomical site cohorts are warranted to validate these initial findings and to further establish the clinical utility of rebound tonometry in tissue expansion.

## Discussion

This pilot study aimed to assess the technical feasibility of employing a rebound tonometer, Icare® TA01i, to measure surface pressure following skin tissue expansion. The Icare® TA01i demonstrated technical success and feasibility by delivering efficient and reliable measurements of skin surface pressure. Importantly, the device did not cause significant complications, including pain, skin damage, infection, or flap necrosis. Finally, the unique design of rebound tonometer presents a promising alternative to intracapsular pressure measurements, potentially allowing for more precise assessments of pressure at specific sites.

Skin tissue expansion is a surgical technique used to generate additional skin for reconstructive purposes (Wagh and Dixit, 2013). The placed dilators are gradually inoculated over time, causing skin stretching and triggering a complex biological response that promotes cellular growth and collagen synthesis (Razzak et al., 2016-). This process allows the skin to accommodate the increased tension resulting from the expansion. The procedure is performed over several weeks until the desired amount of new tissue is achieved. Since the extent of skin stretching directly affects the quality and quantity of the newly formed tissue, meticulous monitoring of dilator pressure is essential for optimal outcomes.

Earlier studies investigating the impact of dilator pressure on skin regeneration predominantly relied on the uniform pressure model (Taber, 1995). This model assumes that the dilator is perfectly compliant and subjected to constant pressure, implying complete contact with no air gap between the dilator and the skin. This approach assesses the effects of pressure on skin expansion based on the total internal pressure of the dilator. PietilÄ et al. proposed that an internal pressure of 30 mmHg (4.0 kPa) is optimal for tissue expansion and new skin generation (PietilÄ et al., 1988). However, subsequent studies revealed discrepancies. Zhao et al. reported that a pressure range of 6.0–8.5 kPa was effective (Wang et al., 2000), while Lu et al. recommended a range of 5.3–8.0 kPa (Lu et al., 2005). These discrepancies may be attributed to the limitations of the uniform pressure model, which does not account for the dilator’s elasticity and geometry. The pressure experienced by the skin differs from the internal pressure of the dilator due to pressure drops across the contact area, influenced by the dilator’s elasticity and geometry. Furthermore, the inherent extensibility of the dilator results in a non-uniform pressure distribution on the skin. Thus, surface pressure measurements may provide a more accurate assessment of pressure effects on skin regeneration. Wu et al. employed an indentation tonometer to measure skin surface pressure with dilators implanted subcutaneously in mice (Wu et al., 2012). Their results indicate the potential to develop a surface pressure measurement system for use in plastic surgery to monitor dilator pressure after each fluid injection, thereby preventing complications arising from excessive local skin expansion.

In this study, we assessed the rebound tonometer for measuring surface pressure. The fundamental principle of the rebound tonometer involves measuring intraocular pressure by assessing the reverse acceleration of the probe after it contacts the cornea. Research shows that rebound tonometers are comparable to indentation tonometers, with no statistically significant differences in performance (Ohana et al., 2017; Jing et al., 2012). The rebound tonometer is equipped with a disposable probe that applies minimal force, thereby causing minimal discomfort to patients and enhancing safety. Our clinical practice did not report any complaints or identify any adverse effects related to the measurement procedure. Furthermore, the rebound tonometer offers several advantages, including portability, no need for topical anesthetics, ease of use, and suitability for use by non-medically trained personnel.

During tissue expansion, the mechanics of the skin dilator system are central to and significantly influence the growth dynamics of the skin. Following inflation, the increased size of the dilator exerts pressure on the surrounding skin, causing deformation to accommodate this pressure. At a specific location, we observe that pressure increases with inflation but decreases as the skin adapts and redistributes the pressure (Figure 3). However, variations in pressure do not always follow this pattern. Inflation does not increase the pressure or accommodation completely eliminates the pressure, and dilator leakage is observed then. Future research will explore the potential correlation between these phenomena. Additionally, from a mechanical perspective, human skin exhibits properties of inhomogeneity, nonlinearity and anisotropy, characterized by *in vivo* pre-stress and the ability to undergo large deformations. Indeed, in our study pressure variations across different locations are non-uniform and reflect the specific conditions at each site (Figure 4).

## Conclusion

This study demonstrated that the rebound tonometer has significant potential for measuring skin surface pressure. In the context of forehead skin expansion, it effectively detects changes in pressure corresponding to variations in dilator size and differences across the expanded area. The device has been shown to be efficient, safe, and reproducible. These results justify further investigation to explore its broader applications in skin reconstruction.

## Data Availability

The original contributions presented in the study are included in the article/Supplementary Material, further inquiries can be directed to the corresponding author.
